# Management and Prognosis of Acute Stroke in Atrial Fibrillation

**DOI:** 10.3390/jcm12175752

**Published:** 2023-09-04

**Authors:** Mette F. Hindsholm, Dorte Damgaard, M. Edip Gurol, David Gaist, Claus Z. Simonsen

**Affiliations:** 1 Department of Neurology, Aarhus University Hospital, 8200 Aarhus, Denmark; dorte.damgaard@auh.rm.dk (D.D.); clausimo@rm.dk (C.Z.S.); 2 Department of Clinical Medicine, Aarhus University, 8200 Aarhus, Denmark; 3 Department of Neurology, Massachusetts General Hospital, Boston, MA 02114, USA; edip@mail.harvard.edu; 4 Harvard Medical School, Boston, MA 02115, USA; 5 Research Unit for Neurology, Odense University Hospital, University of Southern Denmark, 5000 Odense, Denmark; dgaist@health.sdu.dk

**Keywords:** stroke, atrial fibrillation, oral anticoagulation

## Abstract

Atrial fibrillation (AF) is an important risk factor for ischemic stroke (IS). Oral anticoagulation (OAC) significantly reduces the risk of IS in AF but also increases the risk of systemic bleeding, including intracerebral hemorrhage (ICH). AF-related strokes are associated with greater disability and mortality compared to non-AF strokes. The management of patients with AF-related strokes is challenging, and it involves weighing individual risks and benefits in the acute treatment and preventive strategies of these patients. This review summarizes the current knowledge of the acute management of ischemic and hemorrhagic stroke in patients with AF, and the prognosis and potential implications for management both in the acute and long-term setting.

## 1. Introduction

Stroke is a severe and devastating disease affecting more than 12 million people worldwide every year [1]. One of the main risk factors for ischemic stroke (IS) is atrial fibrillation (AF). AF is the most common chronic cardiac arrhythmia, affecting 2–3% of the population in Europe and the USA [2]. AF-related IS are associated with increased mortality, poorer functional outcomes, and increased recurrence rates compared to non-AF-related IS [3]. 

To prevent IS in patients with AF, clinical guidelines recommend long-term oral anticoagulant (OAC) therapy in selected patients [4,5]. OAC therapy reduces the risk of IS by approximately two-thirds [6,7]. However, despite proven efficacy and good compliance, patients with AF may still suffer an IS even when treated with OAC therapy. In addition, long-term OAC treatment also confers an increased risk of hemorrhagic stroke. 

In this review, we summarize the current knowledge in the field of acute stroke in patients with AF regarding stroke characteristics, acute management, and prognosis.

## 2. Ischemic Stroke 

Stroke is a clinical diagnosis defined as the acute onset of focal neurological symptoms of presumed vascular origin. Patients with a stroke can be identified by the typical clinical presentation (sudden hemiparesis, anesthesia, anopia, and loss of function in speech and balance). The scale most often used to evaluate neurologic deficits in the acute setting is the National Institutes of Health Stroke Scale (NIHSS). The NIHSS is composed of 11 items with a score of 0 to 42 (a higher score indicating greater stroke severity) [8]. 

Of all strokes, 85% are ischemic and 15% are hemorrhagic (comprising spontaneous intracerebral hemorrhage (ICH) and atraumatic subarachnoid hemorrhage (SAH)). The clinical presentation is similar among patients with IS and ICH, and brain imaging is needed to distinguish between the two. Brain CT is the imaging modality most commonly used in the acute setting. Here, an ICH will be visualized as a hyperdense, parenchymatous lesion. As brain ischemia may be difficult to visualize acutely on CT; IS is often diagnosed as a combination of a typical clinical syndrome and the absence of bleeding on CT. MRI is superior to CT for providing imaging evidence of brain ischemia and is particularly useful in certain situations, e.g., wake-up stroke and atypical presentations. However, the availability of MRI (and imaging modalities other than CT) is highly setting-dependent, while CT is universally available and can be performed rapidly, including in patients where acute MRI is contraindicated (e.g., patients with pacemakers) or not possible to perform. 

The etiology of an IS has implications for prognosis and long-term management. All patients suffering an IS should be evaluated with brain and neurovascular imaging, cardiac evaluation (rhythm monitoring), and relevant blood tests to establish the etiology of the stroke.

A system for classifying IS according to the underlying stroke etiology has been developed in the Trial of Org 10,172 in Acute Stroke Treatment (TOAST [9]). The TOAST criteria categorize IS into five subtypes based on diagnostic evaluation of brain imaging (CT/MRI), cardiac evaluation (ECG and echocardiography), vascular imaging of cerebral arteries, the results of blood tests, and, in some cases, additional assessments (Table 1). 

Since the cause of IS affects the choice of management, categorizing the stroke etiology according to the TOAST classification is an important step in reducing future risk of stroke. One of the leading pathophysiologic causes of IS is cardioembolism (CE), often secondary to AF. 

## 3. Acute Reperfusion Therapy in Ischemic Stroke

The primary goal in the acute management of IS, no matter the etiology of the stroke, is to minimize brain injury by reestablishing blood flow to the parts of the brain that are ischemic but not yet infarcted. This is achieved through reperfusion therapy. The benefit of reperfusion therapy is time-dependent, and the effect decreases over time. In general, the earlier the treatment is initiated, the better the outcome. 

There are two main options for reperfusion therapy for acute ischemic stroke (AIS): intravenous thrombolysis (IVT) and endovascular treatment (EVT). 

### 3.1. Intravenous Thrombolysis (IVT)

The cornerstone of reperfusion therapy for AIS is IVT with alteplase (a recombinant tissue plasminogen activator, rt-PA) [10]. Alteplase converts plasminogen to plasmin, inducing fibrinolysis of the thrombus [11]. Ten percent of the alteplase dose is administered as an intravenous bolus over one minute, and the rest is infused over one hour, often administered on a pump. 

Randomized controlled trials (RCTs) have shown treatment with alteplase to improve functional outcomes at three months when administered within 4.5 h from stroke onset [12,13,14]. However, treatment with alteplase is associated with a significantly increased risk of hemorrhagic complications, including ICH. The risk of symptomatic ICH (sICH) varies in studies depending on population and definitions, but generally ranges from 2% to 7% in alteplase-treated patients [14,15]. The vast majority of sICH occurs within the first 24 h after IVT administration [16].

The sooner IVT is initiated, the greater the benefit. When administered beyond 4.5 h, harm may exceed the benefit. Hence, it is critical to treat eligible patients as quickly as possible. 

Recently, the drug tenecteplase, an established treatment for acute myocardial infarction, was proven noninferior to alteplase in AIS treatment [17,18,19,20]. Tenecteplase is a modified rt-PA administered as an intravenous bolus with no need for continuous infusion. Bolus administration of tenecteplase seems more advantageous in the setting of AIS management with faster door-to-needle times. For patients with a large vessel occlusion (LVO) who need transfer for EVT at a comprehensive stroke center, work flow and transfer time are greatly improved with tenecteplase. In case of LVO, recent studies have shown treatment with tenecteplase to be associated with increased rates of successful recanalization before initiation of EVT and a better functional outcome at three months compared with patients treated with alteplase [21,22]. This has led most guidelines [23] to recognize tenecteplase 0.25 mg/kg (maximum dose of 25 mg) as an appropriate first-line treatment, preferable to alteplase, in patients eligible for IVT and EVT. 

### 3.2. Endovascular Treatment (EVT)

One in three patients presenting with AIS has an LVO, a blockage of one of the major brain arteries. Different mechanisms may cause LVO: occlusion secondary to intracranial atherosclerosis, artery-to-artery embolization (extracranial atherosclerotic embolism resulting in intracranial occlusion), cardioembolic occlusion (e.g., due to AF), or cryptogenic causes [24]. 

LVO is associated with severe deficits and poor outcomes [25,26]. EVT is indicated in patients with AIS and concomitant LVO. The procedure involves physical extraction of the thrombus through a catheter. Guidelines recommend EVT in patients with LVO presenting within 24 h of stroke onset based on large RCTs [27,28,29,30,31,32,33]. Patients should, if eligible, be treated with IVT even when EVT is considered [34].

## 4. Ischemic Stroke in Atrial Fibrillation

Patients with AF have a five-fold-increased risk of stroke, and approximately one-third of all stroke events are attributable to AF [35,36]. An IS may occur in patients with AF either as the first manifestation of AF (2–4% of patients with AF present with an IS or a transient ischemic attack (TIA)) or despite appropriate OAC treatment. Randomized trials and observational studies report the residual risk of IS in patients treated with OAC to be between 1.4% and 8.9% per year [6,37]. 

**Figure 1:**
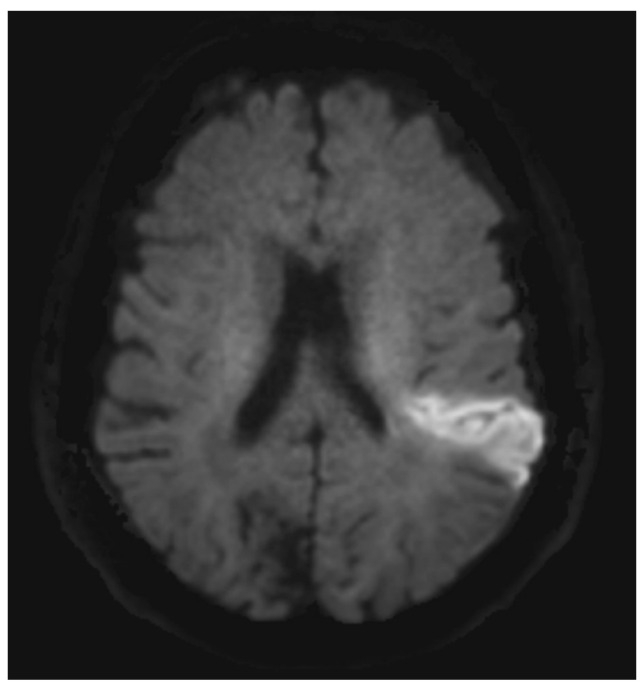
Diffusion-weighted imaging (DWI). Case of cardioembolic infarct in left hemisphere (bright area).

AF-related IS often presents with a distinct radiologic pattern indicating embolic stroke, i.e., a typical wedge-shaped cortical-subcortical infarct [38] (Figure 1). AF-related IS can affect any vascular territory, and multiple vascular territories can be affected [39].

IS in patients with AF has no specific or characteristic clinical features. However, AF-related strokes are often more severe than non-AF-related strokes (i.e., higher NIHSS at admission), presumably due to embolization of larger thrombi [40]. Consequently, patients with AF suffering an IS have worse outcomes compared to patients with sinus rhythm and are at an increased risk of stroke recurrence. This was shown in the Copenhagen Stroke Study [41]: AF-related strokes were associated with higher mortality rates (odds ratio (OR) 1.7; 95% CI, 1.2–2.5), longer hospital stays (50 days vs. 40 days, *p* < 0.001), and lower discharge rates to patients’ own homes versus care facilities (OR 0.60; 95% CI, 0.44–0.85). The Framingham Study showed a similar increase in 30-day mortality in AF-related strokes (OR 1.84; 95% CI, 1.04–3.27) and an increased risk of recurrent stroke during one-year follow-up [42]. These results were exclusively explained by initially more severe strokes and larger infarcts in patients with AF.

Treatment with OAC therapy reduces the risk of IS by 60–70% [43]. OAC therapy has also been shown to reduce stroke severity (lower NIHSS at hospitalization) [44,45], lower the rates of LVO, and result in better functional outcomes at three months after an IS on OAC, compared with AF-related IS in patients not on OAC [46]. For most patients, guidelines recommend the use of direct oral anticoagulant therapy (DOACs, i.e., apixaban, rivaroxaban, edoxaban, or dabigatran) to prevent IS in patients with non-valvular AF (NVAF) rather than vitamin K antagonists (VKA, i.e., warfarin). DOACs perform similarly to warfarin in the prevention of IS, while the risk of treatment-related hemorrhagic stroke is lower when using a DOAC, resulting in decreased mortality [6,47,48,49,50]. 

## 5. Acute Treatment of Ischemic Stroke in Atrial Fibrillation

### 5.1. Use of Intravenous Thrombolysis in Atrial Fibrillation

**Figure 2:**
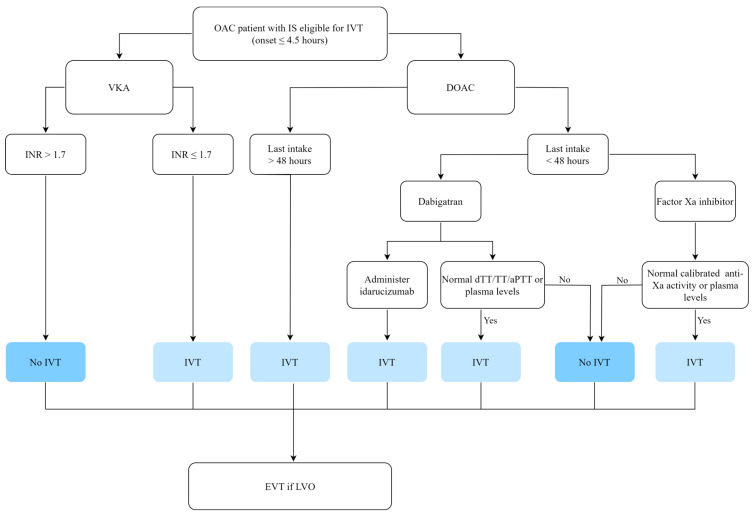
Intravenous thrombolysis (IVT) in patients on oral anticoagulant (OAC) treatment. VKA, vitamin K antagonist; DOAC, direct oral anticoagulants; INR, international normalized ratio; dTT, dilute thrombin time; TT, thrombin time; aPTT, activated partial thromboplastin time; EVT, endovascular treatment.

The initial acute evaluation of patients with AF suffering an IS is similar to the evaluation of non-AF patients. However, the acute management of patients treated with OAC differs from the guidelines described above depending on OAC subtype (Figure 2). 

Recent studies have shown that about 10% of all patients presenting with an AIS are treated with an OAC, with the majority being on a DOAC [51]. Hence, stroke neurologists are often faced with patients with AIS treated with OAC who could be candidates for acute reperfusion therapy. Treatment with OAC therapy is a relative contraindication for IVT. International guidelines recommend that patients treated with VKA and who are otherwise eligible for IVT may be treated with IVT if the INR is 1.7 or less [52,53]. This recommendation is based on register-based observational studies indicating that use of IVT in patients on VKA with an INR of 1.7 or less is not associated with an increased risk of ICH, a decrease in functional outcome, or higher mortality rates at three months [54,55]. 

Recommendations regarding the use of IVT in DOAC patients vary between countries based on different patient selection strategies [56]. The inconsistencies in guidelines on IVT treatment in DOAC patients are the result of current uncertainty regarding monitoring of drug effects in DOAC-treated patients. 

Guidelines recommend against the use of IVT in patients with DOAC intake within 48 h (representing approximately four half-lives of the drugs). This contraindication is based on the presumption of a potential increased risk of ICH. However, data regarding the safety and outcomes of patients treated with IVT while on DOAC to support this presumption are lacking [57].

Information about time since DOAC intake is not always available in the acute setting, and selection of patients based on time since last DOAC intake may lead to the exclusion of IVT-eligible patients who could potentially benefit from this treatment. It is currently debated whether patients can be selected for IVT treatment based on laboratory test results of drug-specific coagulation assays (i.e., anti-Xa activity for Xa inhibitors, thrombin time for dabigatran, or DOAC blood concentrations). In centers with rapid access to calibrated anti-Xa activity levels, selection strategies based on predefined plasma drug-level cut-offs have been adopted [58]. However, several factors complicate the use of such selection strategies, including test availability, turn-around time (analysis time, which varies between 30 and 90 min), a lack of clear cut-off values, and test variability [59]. According to the European Stroke Organisation guideline (ESO, 2021), even with relevant specific anticoagulation tests, there is insufficient evidence to make evidence-based recommendations for this group of patients [52]. 

Another approach for selecting patients on DOAC for IVT is to use reversal agents. There are currently two specific reversal agents (antidotes) approved for DOACs. Only one of them, idarucizumab, is approved prior to IVT. Idarucizumab is approved for the reversal of the anticoagulant effect of dabigatran based on the RE-VERSE AD study [59,60]. Idarucizumab is a humanized monoclonal antibody fragment that binds dabigatran with a high affinity and rapidly neutralizes its anticlotting effect without procoagulant effects. It is quickly administered and, therefore, suitable for use prior to IVT without delaying treatment significantly. Observational studies have found treatment with idarucizumab prior to IVT to be safe and to have similar clinical outcomes and mortality rates as compared to routinely IVT-treated patients, despite an observed delay in door-to-needle time of 22 min in patients treated with the reversal agent [61,62]. According to guidelines, there is still a lack of evidence to make recommendations for or against the combination of idarucizumab and IVT, but several stroke experts recommend this approach [52]. To date, there are no specific reversal agents approved for apixaban, rivaroxaban, or edoxaban prior to IVT treatment. 

Currently, treatment with IVT in DOAC patients is generally withheld due to concerns about treatment safety and the current limited knowledge on the subject. An observational study found that DOAC patients had a five-fold lower rate of IVT treatment than controls not treated with DOACs [46]. 

Newly published observational data indicate that IVT after recent ingestion of DOAC might be safer than expected with lower rates of sICH [57,63,64]. There is ongoing research on this matter (Clinicaltrials.gov NCT02533960).

### 5.2. Use of EVT in Atrial Fibrillation

Approximately half of LVOs are CE, with the majority due to AF [65,66]. The most common sites of occlusion in EVT-eligible patients are the proximal middle cerebral artery (MCA, M1), followed by the more distal part of the MCA (M2) or the intracranial segment of the carotid artery (IC-ICA) [67]. Occlusion of the extracranial segment of the ICA also occurs with or without distal migration of thromboemboli to the IC-ICA segment or M1/M2 segments (artery-to-artery embolization). An observational study found that occlusion of IC-ICA or M1 was associated with AF, higher age, and female sex, whereas tandem occlusion (due to atherosclerosis) was associated with smoking, male sex, and lower age [68]. 

Treatment with OAC is not a contraindication for EVT. Similar rates of EVT have been reported in VKA, DOAC, and non-anticoagulated patients, with no significant time delay in symptom onset to groin puncture in VKA and DOAC patients compared to controls [46]. Current guidelines recommend treatment with IVT prior to EVT in eligible patients. However, a meta-analysis comparing direct EVT to IVT followed by EVT has shown similar safety and functional outcomes in the two groups [69]. Hence, DOAC patients with a concomitant LVO presenting directly to an EVT center should preferably bypass IVT evaluation and start EVT directly to minimize delay. 

Only very few anticoagulated patients participated in randomized trials of EVT [32,33], and data regarding the safety and efficacy of EVT in OAC-treated patients rely mainly on observational studies. These studies have shown similar rates of recanalization in anticoagulated as in non-anticoagulated (OAC-naïve) patients, and OAC status has not been associated with ICH [70,71,72]. Current guidelines do not distinguish between VKA or DOAC prior to EVT. However, two large meta-analyses from 2020 and 2022 found VKA treatment associated with a higher risk of sICH and mortality after EVT compared to OAC-naïve patients [73,74]. This association was not found in DOAC patients. A newly published register-based study found recent use of VKA to significantly increase the risk of sICH (8.3%) after EVT if INR was higher than 1.7, compared with OAC-naïve patients [75]. DOAC treatment has also been associated with increased rates of successful reperfusion and better functional outcomes after EVT compared to VKA [76].

Some thrombi are easier to retrieve during EVT than others, and successful reperfusion is achieved in 44% to 88% of cases [77]. The reasons for the differences in reperfusion rates are multifaceted. Thrombus composition may influence the efficacy of EVT. Studies indicate that red blood cell-rich thrombi are associated with higher recanalization rates compared to fibrin-rich thrombi, which have a higher friction and therefore stronger interaction with the vessel wall [78,79]. Histopathological differences in thrombi composition have also been described as being associated with stroke etiology [66]; however, whether this is the case is highly debated. 

CE-caused LVOs have been associated with higher EVT efficacy due to faster and higher rates of successful reperfusion, potentially leading to better clinical outcomes compared to large artery atherosclerotic (LAA) LVOs [66,68,80]. LAA LVOs have also been found to have higher mortality rates, probably due to the complexity of the procedure and higher rates of re-occlusion in this group of patients [81]. These findings might be related to thrombus composition and etiologic differences in the extent of atherosclerosis and vessel elongation/tortuosity, factors that may impede access to the occlusion. However, other studies did not find any differences in rates of successful reperfusion or clinical outcomes in CE LVOs compared to other etiologies [81]; some even described worse outcomes in CE LVOs [82]. 

## 6. Determining the Cause of Ischemic Stroke in OAC-Treated Patients

IS despite OAC, often referred to as “OAC failure”, comprises heterogeneous etiologies. OAC failure may be caused by non-compliance, insufficient anticoagulation (off-label low DOAC dose or subtherapeutic VKA dosage), CE stroke despite OAC (due to advanced or other cardiac disease), or other stroke mechanisms (e.g., large vessel atherosclerosis, small vessel disease, malignancy). A recent analysis of 1674 patients with IS despite OAC therapy found stroke etiology in patients on DOAC/VKA to be CE in 49%/37%, poor adherence or insufficient dose in 23%/43%, and a competing mechanism in 28%/20%, respectively [83]. The RENo study, comparing patients with AF who suffered an IS while on DOAC versus those who did not, found off-label low DOAC dose, atrial enlargement, hyperlipidemia, high CHA_2_DS_2_-VASc score, and increased AF burden to be associated with IS despite OAC treatment [84]. Hence, all patients with AF who suffer an IS while treated with OAC should have a thorough workup to determine the most likely stroke mechanism. 

In 20–30% of OAC failures, a competing stroke mechanism is identified. Generally, OAC failures require life-long OAC therapy even when other etiologies, such as large or small vessel disease, are identified. However, some more rare causes of IS indicate specific antithrombotic strategies (i.e., antiphospholipid antibody syndrome, hypercoagulability (malignancy), or infective endocarditis) [58].

## 7. Restarting OAC after Ischemic Stroke

OAC treatment is initially withheld after admission to reduce the risk of hemorrhagic transformation (HT), a potential complication of AIS often occurring within the first few days after stroke onset [85]. Symptomatic HT has been reported in 2% to 20% of patients in RCTs [86], and it is associated with increased stroke mortality and morbidity. 

Risk factors for HT include stroke severity (large infarct size), reperfusion treatment (both IVT and EVT), hypertension, hyperglycemia, prior OAC treatment, and age [87]. Due to the concern of a possible association between early initiation of anticoagulation and an increased risk of HT, OAC therapy is withheld for days to weeks after IS in AF patients. But the risk of recurrent IS is also increased within the first few days to weeks after an IS. An observational study of AF-related IS reported the risk of recurrent IS to vary between 0.5% and 1.3% per day within 14 days of admission [88]. The clinical conundrum is when to (re)start OAC to balance these risks.

In patients treated with IVT, guidelines recommend withholding anticoagulants and antiplatelet agents for at least 24 h after treatment and until repeated neuroimaging confirms no bleeding to mitigate the risk of hemorrhagic complications. Guideline recommendations regarding timing OAC after an IS have varied due to the dearth of high-quality evidence on the subject. OAC initiation has generally been guided by the size of the infarct; the more severe the stroke, the later the initiation of OAC. The 1–6–12 day rule, introduced in 2013 by the European Heart Rhythm Association of the European Society of Cardiology (EHRA-ESC) [89], has been the most prevailing strategy regarding OAC initiation after IS. The severity of the stroke is based on the NIHSS score; the higher the score, the more severe the stroke. This strategy has been adopted in various associations, including the European Stroke Organisation guideline [90], with small variations (3–4 days after mild stroke, 7 days after moderate stroke, and 14 days after major infarcts). The 2021 AHA/ASA guideline [90] recommends delaying initiation of OAC beyond 14 days in patients at high risk of HT to reduce the risk of ICH, whereas patients at low risk of HT may initiate OAC after 2 to 14 days to reduce the risk of recurrent IS. These recommendations are based on expert opinions, not RCT data.

Treatment with DOAC is recommended in preference to VKA in patients with NVAF and previous IS/TIA [90,91]. 

The ELAN trial, an RCT published in 2023, investigated the effect of early initiation (within 48 h after mild or moderate strokes and 6–7 days after major strokes) versus later initiation (according to current guidelines: 3–4 days after minor stroke, 6–7 after moderate stroke, and 12–14 after major stroke) of DOAC in patients with AF and IS [92]. The study used imaging-based classification of stroke severity (i.e., minor, moderate, or major), and not NIHSS score as in current guidelines and did not include patients with OAC use prior to their IS. 

**Figure 3:**
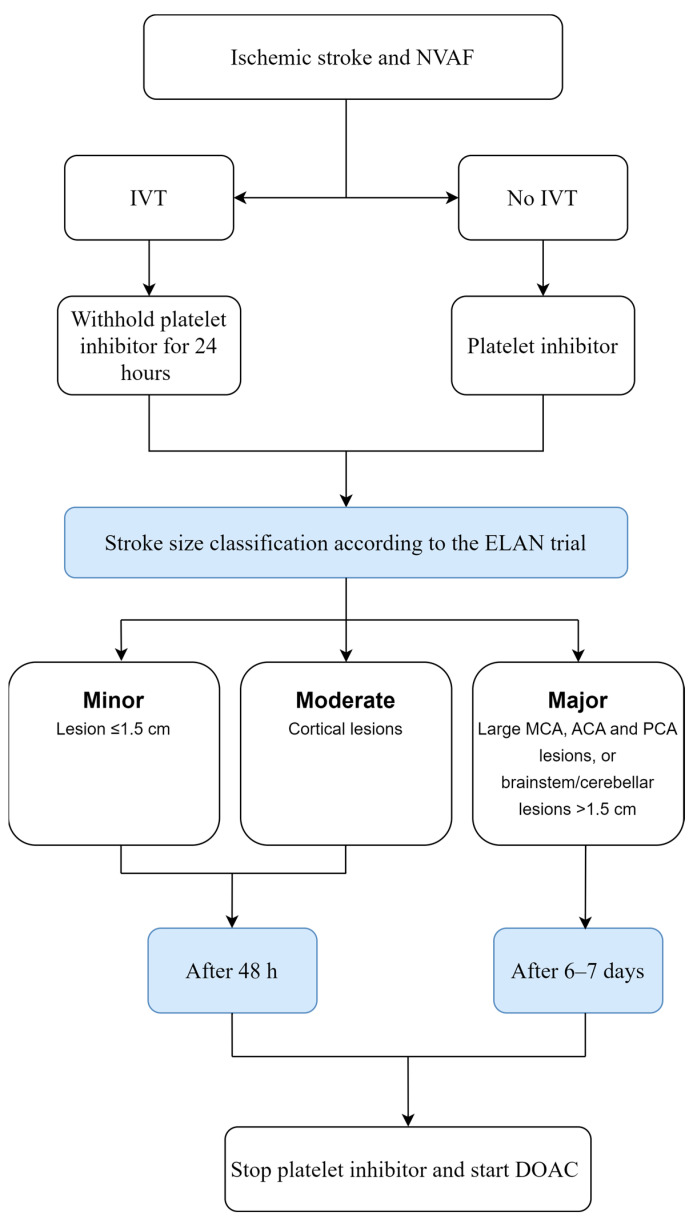
Timing of initiation of oral anticoagulation (OAC) after ischemic stroke (IS) in patients with non-valvular atrial fibrillation (NVAF) according to the ELAN trial results [92]. IVT, intravenous thrombolysis; MCA, middle cerebral artery; ACA, anterior cerebral artery; PCA, posterior cerebral artery; DOAC, direct oral anticoagulants.

The study found the incidence of recurrent IS within 30 days to be 1.4% in the early-treatment group vs. 2.5% in the later-treatment group (OR 0.57; 95% CI, 0.29–1.07), while ICH occurred at the same rate in both arms (0.2%). The similar TIMING trial, published in 2022, found early initiation (within 4 days) noninferior to delayed start (within 5–10 days) of DOACs after IS, with no ICH in any of the groups [93]. The results of these studies, along with the promising results of observational studies of DOAC initiation after IS [94,95,96], indicate that early DOAC initiation is not associated with any safety concerns. The decision making regarding the timing of OAC initiation after an IS always has to be individually assessed based on risk factors, but according to the ELAN results, early treatment with DOAC should be considered for secondary prevention in AF after an IS (Figure 3). 

## 8. Prognosis

OAC failure is associated with an increased mortality rate. An analysis based on combined data from the pivotal DOAC randomized trials (the COMBINE AF dataset [97]) found all-cause mortality particularly high in the early time period following IS (cumulative incidence of 12.4% (95% CI, 10.5–14.4%) at three months and 18.1% (95% CI, 15.7–20.4%) at one year [98]). The observed one-year post-stroke mortality rate in OAC failures was higher than the baseline mortality rate of patients treated with OAC in the individual trials. 

The risk of stroke recurrence (ischemic or hemorrhagic) in AF patients suffering an IS is associated with high CHA_2_DS_2_-VASc score, high NIHSS score at admission, large infarct size, and type of anticoagulation used after index IS (i.e., higher for VKA than DOAC) [99]. 

Prior IS or TIA seems to be one of the strongest prognostic factors for future IS. Results from subgroup analysis from five major DOAC trials in AF (RE-LY [100], AVERROES [101], ROCKET AF [102], ARISTOTLE [103], and ENGAGE AF-TIME [104]) suggest that the risk of recurrent IS is doubled in patients with prior IS or TIA (relative risk (RR) 1.8–2.9 per 100 patient years in patients with prior IS/TIA vs. 0.7–1.4 per 100 patient years in patients with no prior IS/TIA) [98]. Hence, prior IS or TIA is a central component in stroke-risk scoring systems of AF patients and confers a strong recommendation for lifelong OAC therapy for secondary stroke prevention (a CHA_2_DS_2_-VASc score of at least two).

An analysis based on combined data from the pivotal DOAC randomized trials (the COMBINE AF dataset [97]) found the risk of recurrent IS to be particularly high in OAC-failure patients [98]. The cumulative incidence of recurrent IS in OAC failures was 7.0% (95% CI, 5.2–8.7%) at one year and 10.3% (95% CI, 7.8–2.8%) at two years. The incidence was particularly high within the first months (3.0% (95% CI, 1.9–4.0%) at 3 months). An Asian observational study reported similar rates (cumulative incidence of 5.3% in patients with OAC failure vs. 2.9% in patients without prior OAC treatment (HR 1.50 (95% CI, 1.02–2.21)) [105]. Observational studies are in line with these findings [37,106]. These results indicate that OAC-failure patients might have unknown risk factors that increase their future risk of recurrent IS. 

Management of OAC failure is challenging, and there are currently limited data on how to guide treatment of this group of patients. Strategies include strict risk factor control (e.g., hypertension, diabetes mellitus, and dyslipidemia) and continuous OAC treatment. Data on the effect of switching anticoagulant type (VKA to DOAC, DOAC to VKA, or between DOACs) to reduce the future risk of recurrent IS is limited. Most observational studies have not found switching between OACs to be associated with a lower risk of recurrent IS [37,106]. One study found a treatment with DOAC after index IS to be associated with lower odds for recurrent IS compared to VKA [83]. However, there are no data from randomized trials to support the strategy of switching between OACs.

There is no proven benefit in the reduction of recurrent IS or death from adding platelet inhibitors on top of OAC therapy, but only an increased risk of bleeding complications, including ICH [83,107,108].

Whether left atrial appendage occlusion (LAAO) plus OAC treatment could be beneficial in patients with AF-related IS despite OAC treatment is currently unknown. The LAAOS III trial, where patients with AF and OAC treatment were randomized to LAAO or no LAAO during open cardiac surgery, found a 30% lower rate of stroke or systemic embolisms in the occlusion group compared to the no-occlusion group (4.8% vs. 7.0%, HR 0.67; 95% CI, 0.53–0.85) [109]. Whether these results are transferable to percutaneous LAAO is uncertain. In patients with prior IS or TIA and an absolute contraindication to OAC, LAAO may be considered according to guidelines [110]. 

## 9. Hemorrhagic Stroke

ICH is the second most common cause of stroke, accounting for approximately 10% of all strokes but 50% of stroke mortality. It is associated with a 30-day mortality rate of 40–50%, and only about 30% of ICH patients gain functional independence after 90 days [111]. 

**Figure 4:**
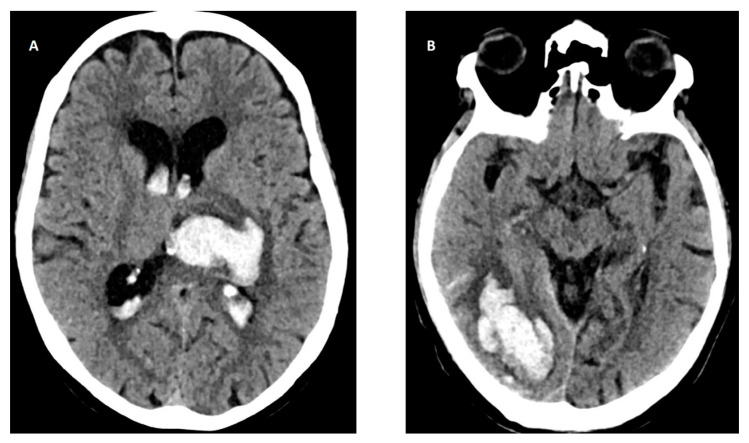
Head CT of intracerebral hemorrhage (ICH). (**A**) Deep basal ganglia ICH in the left hemisphere with intraventricular hemorrhage (IVH) due to hypertension. (**B**) Lobar ICH in right occipital lobe with subarachnoid hemorrhage (SAH) probably due to cerebral amyloid angiopathy (CAA).

The most common cause of ICH is small vessel disease due to hypertension or cerebral amyloid angiopathy (CAA), a neurodegenerative age-dependent disorder characterized by amyloid deposition in the small cortical and leptomeningeal arteries, with an increased risk of IS and ICH [112]. Other causes include hemorrhage due to sinus thrombosis, rupture of vascular malformations (i.e., arteriovenous malformations, cavernous angiomas, and cerebral aneurysms), or neoplasms [113]. The location of ICH is associated with its etiology. In spontaneous (non-traumatic) ICH, lobar hematoma location may be indicative of underlying CAA, whereas non-lobar hemorrhages (basal ganglia, thalamus, and pontine) are almost always caused by hypertensive arteriopathy (Figure 4). 

Risk factors for ICH include increasing age (incidence rate of 0.1% in people below age 45 vs. 9.6% for people over age 85 [114]), hypertension (which doubles the risk of ICH [115,116]), and use of OAC. 

The majority of patients suffering an ICH present with elevated blood pressure (BP) caused by pain, increased intracranial pressure (ICP), or untreated baseline hypertension. Clinically, ICH presents with similar symptoms as IS. However, some symptoms may indicate the presence of ICH rather than IS. These include progressive worsening of symptoms, severely elevated blood pressure, seizures, and signs of elevated intracranial pressure (ICP) (headache, nausea, vomiting, and decreased level of consciousness). 

## 10. Intracerebral Hemorrhage in Atrial Fibrillation

ICH is the most feared and devastating complication in AF patients treated with OAC. Warfarin is associated with a two- to five-fold increased risk of ICH, whereas the risk is lower in DOAC therapy (relative risk reduction of about 50% compared to warfarin [117]). 

OAC treatment in ICH patients is associated with greater hematoma growth, neurologic deterioration, and increased risk of death and poor outcome [118]. OAC-related ICH accounts for 25% of all ICH cases, with a high case fatality rate of 52% vs. 25% in non-OAC ICH [119].

The prevalence of OAC-related ICH is increasing as AF prevalence [120] and prescriptions of OAC therapy are increasing [121]. 

## 11. Acute Treatment of ICH

The acute treatment of ICH consists of BP lowering, reversion of possible hypercoagulable state, possible surgery, and general stroke care. A recent RCT showed that implementation of a care bundle protocol including early intensive systolic BP (SBP) lowering (<140 mmHg), strict glucose control, antipyretic treatment, and reversal of warfarin-related anticoagulation within one hour improved functional outcomes in ICH patients [122]. 

### 11.1. Medical Treatment

Guidelines recommend lowering systolic BP to a target of 140 mmHg (130–150 mmHg) in the acute phase [123,124]. This recommendation is based on two large RCTs of BP-lowering strategies in patients with acute ICH (INTERACT2 [125] and ATACH-II [126]). A pooled analysis from these studies found that each reduction in systolic BP of 10 mmHg within the first 24 h was associated with a 10% increase in odds of a better functional outcome (down to a threshold of 120 to 130 mmHg) [127]. Lowering BP too much (i.e., below 130 mmHg) or too fast may be associated with a worse outcome [128] and increases the risk of adverse events like cerebral hypoperfusion and kidney injury. Elevated BP should be treated with drugs with short half-lives (such as labetalol or nicardipine) for better BP control and to avoid hypotension. 

### 11.2. Surgical Treatment

The role of surgical treatment in patients with ICH has been intensively debated over the years. There is still a lack of evidence regarding which patients might benefit from surgical treatment, the appropriate surgical technique, and the optimal timing of intervention. Overall, RCTs evaluating the neurosurgical evacuation of hematomas have not shown improved functional outcomes of surgery compared with standard medical treatment [129,130]. However, some patients with signs of elevated ICP (neurologic deterioration), especially patients with fossa posterior hemorrhages, may benefit from surgical treatment, and in certain cases, guidelines recommend surgery to reduce mortality [123].

A recent study (ENRICH [131], ClinicalTrials.gov NTC02880878), results not yet published, comparing early minimally invasive surgery (MIPS) and standard medical management in primary, supratentorial ICH is the first RCT to demonstrate a positive effect on mortality and functional outcome in patients treated with early surgery. There are currently several ongoing trials investigating this correlation. 

### 11.3. Treatment in Anticoagulated Patients

**Figure 5:**
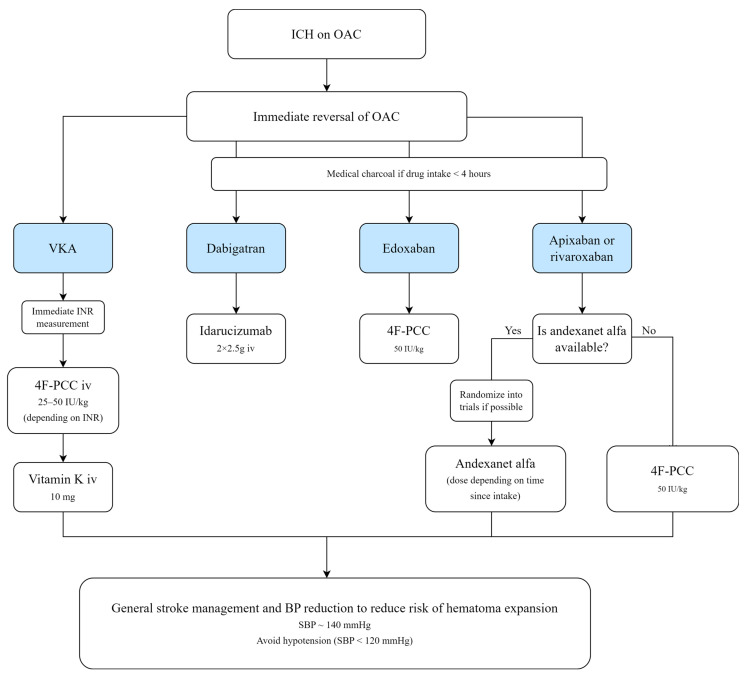
Reversal of oral anticoagulation (OAC) in patients with intracerebral hemorrhage (ICH). VKA, vitamin K antagonist; 4F-PCC, f-factor prothrombin complex concentrate; BP, blood pressure; SBP, systolic blood pressure.

Treatment of OAC-related ICH in AF includes discontinuation of anticoagulation and immediate reversal of anticoagulation to reduce the risk of hematoma expansion. The choice of reversal strategy depends on the OAC type (Figure 5). 

VKAs are recommended to be reversed with a 4-factor (factor II, VII, IX, and X) prothrombin complex concentrate (4F-PCC). Since the half-life of 4F-PCC is shorter than the half-life of VKA, it is recommended to also administer intravenous vitamin K. If not available, fresh frozen plasma (FFP) may be administered instead of 4F-PCC. This recommendation is based on the INCH trial, which showed faster INR normalization and smaller hematoma expansion with 4F-PCC compared to FFP [132]. However, despite effective reversal of warfarin in the acute setting, mortality is still high in warfarin-related ICH patients (up to 42% in-hospital mortality has been reported [133]). 

There is only limited data on the efficacy of specific and unspecific approaches to reverse DOACs, and RCTs in DOAC-related ICH are lacking. Guidelines recommend the use of specific reversal agents (i.e., idarucizumab and andexanet alfa) for the reversal of DOACs if available, and PCC is recommended when not available [134]. 

In patients treated with dabigatran, idarucizumab should preferably be administered to reverse the anticoagulant effect. Based on the REVERSE-AD study, idarucizumab seems safe and has a fast reversal effect, according to coagulation tests [60]. However, data are lacking to evaluate the effect of idarucizumab on clinical outcomes. 

Andexanet alfa has been shown to reverse the anticoagulant activity of factor Xa inhibitors [135,136]. It is approved for the reversal of rivaroxaban and apixaban in patients with uncontrolled, life-threatening bleeding. Andexanet alfa is a modified recombinant factor Xa that binds factor Xa inhibitors, reducing their anticoagulant effects [137]. It is administered as a bolus followed by a two-hour infusion. Serial plasma concentration measurement is recommended after administration to observe for potential rebound effects [110]. Most recently, a phase IV RCT, ANNEXA-I, was stopped early. It showed superior hemostatic efficacy of andexanet alfa compared to usual care in limiting expansion of ICH (results not yet published, ClinicalTrials.gov NCT03661528). Data on the safety and clinical outcomes of andexanet alfa treatment are awaited, and guidelines recommend randomizing into trials if possible [134]. 

## 12. Restarting Anticoagulation Therapy in Atrial Fibrillation after Intracerebral Hemorrhage

Optimal timing of OAC resumption after ICH in NVAF is often a clinical dilemma. The decision requires balancing future hemorrhagic and thromboembolic risks at an individual level. Guidelines recommend enrollment in ongoing randomized trials investigating optimal therapeutic management after ICH in AF patients. 

The risk of IS often exceeds the risk of re-ICH in AF patients, with risk–benefit analyses favoring resumption of OAC therapy after bleeding has resolved [138]. Resumption of OAC has been associated with a lower risk of thromboembolic events, lower rates of mortality, and no increase in re-ICH risk in meta-analyses of OAC-related ICH [139,140,141]. In a large meta-analysis of AF patients, the association of OAC resumption with decreased mortality and all-cause stroke was also observed regardless of hematoma location (lobar vs. non-lobar) [142]. A 2023 Cochrane review on antithrombotic treatment after ICH in patients with AF found OAC initiation after ICH to reduce IS events but probably to increase the risk of ICH (based on three RCTs) [143]. 

The risk of ICH and re-ICH is increased in CAA patients [144]. This has often led clinicians to abstain from OAC treatment in AF patients with concomitant CAA to mitigate ICH risk. However, the presence of AF might confer an even higher risk of IS and mortality that outweighs the risk of re-ICH in this group of patients. The evidence and knowledge on CAA-related ICH and resumption of OAC are limited. OAC therapy may be indicated in CAA-related lobar hemorrhages depending on imaging findings (presence of hemorrhagic MRI markers on blood-sensitive sequences: cerebral microbleeds (CMBs) and degree of cortical superficial siderosis (cSS)) and in patients with well-controlled BP [110]. 

Individual decision-making regarding resumption of OAC treatment after ICH should include an evaluation of the following risk factors: BP control, age, ICH location, burden of small vessel disease (CMBs, leukoaraiosis, and cSS), and indication of antiplatelets. Data suggestive of individual factors favoring OAC resumption may include younger age, deep ICH location, and well-controlled hypertension [110]. The available evidence regarding risk factors in OAC resumption is scarce, highlighting the necessity of identifying sub-groups for whom resuming OAC therapy may be more beneficial or harmful in future studies.

In the absence of randomized data, the optimal time to resume OAC treatment is unknown. Timing depends on OAC indication and individual risk–benefit analyses. In general, restarting OAC in patients with NVAF seems safe within four to eight weeks after index ICH [110]. An observational study, limited by selection bias, found OAC initiation within seven to eight weeks after index ICH to optimize treatment benefit and minimize risks [145]. Treatment with DOAC therapy is preferred over VKA in ICH survivors with NVAF due to DOAC’s reduced risk of bleeding.

In OAC-related ICH, patients with especially high bleeding risks or other contraindications to OAC treatment may be considered for LAAO [123]. 

## 13. Prognosis and Risk of Recurrent ICH

Less than 50% of patients suffering an ICH are alive within one year, and only 30% within five years [146]. Increasing age, low GCS, large ICH volume, presence of intraventricular hemorrhage, and deep or infratentorial ICH are all factors associated with increased mortality. 

The risk of recurrent ICH is especially high within the first 12 months. A meta-analysis investigating the long-term prognosis of ICH found the annual risk of re-ICH to vary between 1.3% and 7.4% [146]. 

Hypertension is consistently the primary risk factor associated with an increased risk of re-ICH and predisposes to both lobar and non-lobar bleeding [147]. Thus, aggressive management of BP is essential in the prevention of re-ICH [148,149]. 

The location and etiology of ICH are associated with a higher risk of re-ICH, probably due to the underlying arteriopathy [144]. The risk of recurrent bleeding is higher in lobar bleedings (suggestive of CAA), with an annual risk of 3.7% to 5.1%, compared to non-lobar bleedings (associated with hypertension), with an annual risk of 1.2% to 1.8% [150,151]. 

**Figure 6:**
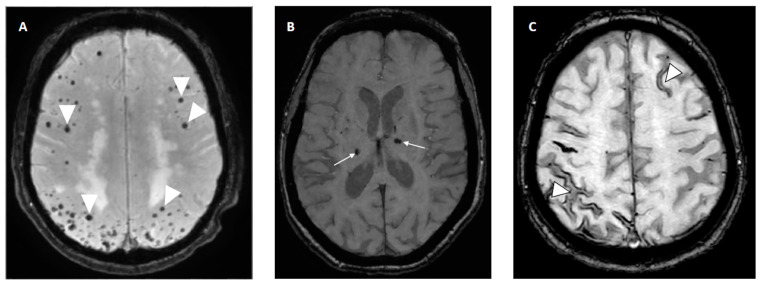
Radiologic bleeding markers on susceptibility weighted imaging (SWI) on MRI. (**A**) Multiple lobar cerebral microbleeds (CMBs) in patient with cerebral amyloid angiopathy (CAA). (**B**) Deep CMBs related to hypertension. (**C**) Cortical superficial siderosis (cSS) in frontal left hemisphere and posterior right sulci in patient with CAA.

The presence of CMBs, a radiologic biomarker of small vessel disease in the brain visible on MRI blood-sensitive sequences (SWI or T2*) as small, black dots (accumulation of blood products), has also been related to the risk of re-ICH. CMBs are more often present in patients with re-ICH compared to patients with first-ever ICH (83% vs. 52% [152]), and their presence may predict an increased risk of ICH [144]. The location of CMBs may indicate the underlying arteriopathy: deep CMBs are related to hypertensive arteriopathy, whereas strictly lobar CMBs are more likely associated with CAA [153] (Figure 6). As the risk of re-ICH is significantly higher in lobar CAA-related ICH, lobar CMBs indicative of CAA may be a stronger ICH predictor than deep (hypertensive) CMBs [153].

The presence of cSS, the deposition of iron on the brain surface with characteristic curvilinear low signal on MRI (SWI and T2* sequences) along the cerebral convexities due to prior bleeding, is an MRI biomarker of CAA [154] (Figure 6). The presence of cSS has been shown to increase the risk of recurrent ICH [155]. It may be the most potent independent marker of increased risk for CAA-related ICH, especially if disseminated (involving more than three sulci) [110,156]. A cohort study evaluating patients with CAA-related ICH reported increased re-ICH rates with increasing degree of cSS [157]. 

## 14. Conclusions

Stroke in atrial fibrillation is a common and severe condition. OAC therapy reduces the risk of IS, but in spite of relevant OAC treatment, patients with AF may still suffer an IS. OAC patients are also at an increased risk of ICH, a condition more severe than ICH off OAC. Both acute and long-term treatment of patients with AF and IS or ICH represents a common clinical challenge. 

Future studies including robust clinical trials regarding the use and safety of IVT in DOAC patients, reversal strategies of DOAC in the acute setting of stroke (ischemic and hemorrhagic), optimal timing of OAC after IS and ICH, identification of high-risk stroke patients, and optimal treatment of OAC failures are needed. 

## Figures and Tables

**Table 1:** TOAST classification of subtype of ischemic stroke.

Etiology	Definition	CT/MRI Scan
Large artery atherosclerosis (LAA) (embolus/thrombus) *	Imaging showing significant stenosis (>50%) of ipsilateral extra- or intracranial artery or occlusion of major brain artery or branch cortical artery due to atherosclerosis.	Cortical and cerebellar lesions, and brain stem and subcortical infarcts ≥ 1.5 cm in diameter.
Cardioembolism *	Identification of at least one cardiac high-risk/medium-risk ** source for embolism.	Cortical and cerebellar lesions, and brain stem and subcortical infarcts ≥ 1.5 cm in diameter.
Small-vessel occlusion (lacune) *	Clinical lacunar syndromes. Absence of LAA and cardioembolic source identified. Comorbidity of diabetes mellitus and hypertension supports etiology.	Brain stem and subcortical lesions ≤ 1.5 cm in diameter. OR No detectable lesion.
Stroke of other determined etiology *	Identification of rarer causes of stroke (vasculitis, hypercoagulable states, and hematologic disorders).	Visual infarct regardless of size and location.
Stroke of undetermined etiology	Not possible to determine cause of stroke with confidence due to: ≥2 causes are identified Negative evaluations Incomplete evaluations	

* Possible or probable. ** High-risk/medium-risk sources of cardioembolism are based on the evidence of their propensities for embolism [9].

## Data Availability

Not applicable.

## Abbreviations

AF	Atrial fibrillation
AIS	Acute ischemic stroke
BP	Blood pressure
CAA	Cerebral amyloid angiopathy
CE	Cardioembolism
CMB	Cerebral microbleed
cSS	Cortical superficial siderosis
DOAC	Direct oral anticoagulants
EVT	Endovascular treatment
HT	Hemorrhagic transformation
ICH	Intracerebral hemorrhage
IS	Ischemic stroke
IVT	Intravenous thrombolysis
LAAO	Left atrial appendage occlusion
LVO	Large vessel occlusion
NIHSS	National Institutes of Health Stroke Scale
NVAF	Non-valvular atrial fibrillation
OAC	Oral anticoagulation
rt-PA	Recombinant tissue plasminogen activator
SAH	Subarachnoid hemorrhage
sICH	Symptomatic intracerebral hemorrhage
TIA	Transient ischemic attack
VKA	Vitamin K agonist
